# A meta-analysis of neoadjuvant chemotherapy versus neoadjuvant chemoradiotherapy for locally resectable esophageal cancer based on RCTs

**DOI:** 10.3389/fonc.2026.1728150

**Published:** 2026-02-12

**Authors:** Tinghui Xu, Lei Wang, Xiaoming Zhang, Xinyan Yu, Shiyan Zhang, Jiamu Zheng, Jie Fei

**Affiliations:** 1 Department of Cardiothoracic Surgery, Ningbo Hospital of Integrated Traditional Chinese and Western Medicine, Ningbo, Zhejiang, China; 2 The Second Clinical Medical College, Zhejiang Chinese Medical University, Hangzhou, Zhejiang, China; 3 The First Clinical Medical College, Zhejiang Chinese Medical University, Hangzhou, Zhejiang, China

**Keywords:** chemoradiotherapy, chemotherapy, esophageal cancer, meta-analysis, neoadjuvant, resectable

## Abstract

**Background:**

For locally resectable esophageal cancer, the efficacy of neoadjuvant chemotherapy (NCT) and neoadjuvant chemoradiotherapy (NCRT) remains controversial. Therefore, this study explores the efficacy difference between NCT and NCRT in locally resectable esophageal cancer (including gastroesophageal junction cancer).

**Methods:**

This study adhered to the guidelines of the PRISMA (Preferred Reporting Items for Systematic Reviews and Meta-Analyses) statement and was prospectively registered on PROSPERO with the registration number CRD420251044577. Four electronic databases—PubMed, Web of Science, Cochrane Library, and ClinicalTrials.gov—were searched from their inception to May 2025. The primary outcomes of this study were overall survival (OS) and progression-free survival (PFS), which were presented as hazard ratios (HRs) and 95% confidence intervals (CIs). A random-effects model was uniformly used for the pooled analysis and leave-one-out sensitivity analysis and publication bias detection were conducted.

**Results:**

After searching and screening, six randomized controlled trials (RCTs) were finally included, involving 886 patients in the experimental group (NCT group) and 877 patients in the control group (NCRT group). The results of statistical analysis show no statistically significant difference in OS (HR = 1.06, 95%CI: 0.85-1.31; p=0.603, I²=59.53%, τ²=0.0411) and PFS (HR = 1.05, 95%CI: 0.79-1.40; p=0.718, I²=76.38%, τ²=0.0892) between NCT and NCRT. Sensitivity analysis reveals that the results remain stable when each study is excluded one by one, and no publication bias is detected using Begg’s test.

**Conclusion:**

The results of this study suggest that in the perioperative management of locally resectable esophageal cancer (including gastroesophageal junction cancer), existing data do not yet demonstrate a significant survival advantage for NCRT over NCT. Individualized decision-making is therefore recommended.

## Introduction

According to the latest epidemiological data, esophageal cancer ranks as the seventh leading cause of cancer-related death globally and the 11th most common in terms of new cases (1). In 2022, new esophageal cancer cases exceeded 510,000 (age-standardized rate (ASR) of 4.26 per 100,000), with deaths surpassing 440,000 (2). Pathologically, esophageal cancer primarily comprises esophageal squamous cell carcinoma (ESCC) and esophageal adenocarcinoma (EAC), while esophagogastric junction cancer is predominantly EAC. The highest incidence rates for EAC occur in Northern Europe (ASR 3.5) and North America (ASR 2.2), whereas Asia exhibits the highest incidence of ESCC, accounting for over 80% of global ESCC cases (3). Esophageal cancer is prone to relapse after treatment, and the recurrence mostly occurs within 2 years after completion of local therapy, with a recurrence rate of more than 85% (4, 5).

Current treatment modalities for esophageal cancer include surgery, chemotherapy, radiotherapy, and immunotherapy, as well as their combination. Even with multimodal therapy, the prognosis for esophageal cancer remains poor, with a 5-year survival rate of only 50%-60% (6). For patients with locally advanced, unresectable disease, guidelines from the American Society of Clinical Oncology recommend definitive chemoradiotherapy. For locally resectable esophageal cancer, guidelines suggest preoperative chemoradiotherapy or perioperative chemotherapy (7). Immunotherapy is recommended for patients with residual disease after neoadjuvant chemoradiotherapy (NCRT) and surgery (8).

Neoadjuvant therapy refers to systemic treatment administered before definitive local therapy for solid tumors. It aims to reduce tumor volume, eliminate micrometastases, and downstage the disease, thereby creating more favorable conditions for subsequent local treatment. Accumulating evidence indicates that both neoadjuvant chemotherapy (NCT) and NCRT improve perioperative survival and reduce recurrence in patients with esophageal cancer (5, 9). It was previously widely accepted that NCRT was superior to NCT in efficacy. However, several recent studies suggest that there is no significant difference in efficacy between NCT and NCRT (6, 10). Furthermore, published systematic reviews and meta-analyses have yielded varying conclusions, indicating that the controversy on this issue remains unresolved (11, 12). Therefore, this study conducts a meta-analysis to compare the efficacy of NCT and NCRT in locally resectable esophageal cancer (including gastroesophageal junction cancer).

## Methods

### Search strategy

This meta-analysis adhered to the PRISMA (Preferred Reporting Items for Systematic Reviews and Meta-Analyses) statement guidelines for reporting systematic reviews and meta-analyses (13). It was prospectively registered on the PROSPERO website under registration number CRD420251044577. To identify clinical trial articles investigating the efficacy of NCT versus NCRT for esophageal cancer (including gastroesophageal junction cancer), a systematic review was conducted in four electronic databases: PubMed, Web of Science, Cochrane Library, and ClinicalTrials.gov. Search terms were a combination of mesh subject words and free words: “(Esophageal Cancer OR Gastroesophageal Junction Cancer) AND (Neoadjuvant OR Preoperative OR Perioperative) AND Chemoradiotherapy AND Chemotherapy”. The specific search terms were adapted to suit the unique features of each database. To minimize the risk of missing relevant studies, the authors manually searched the reference lists of included articles. The search encompassed the period from the inception of each database until May 2025 and was restricted to articles published in the English language. Two independent researchers performed all search processes. Any disagreements arising during the process were resolved through discussion and consensus between the researchers or, if necessary, by consultation with a third reviewer.

### Inclusion and exclusion criteria

Inclusion criteria were defined according to the PICOS principle. Only studies that met all of the following inclusion criteria were considered for inclusion:

1. Participants comprised patients with locally resectable esophageal cancer, including ESCC or EAC of the upper, middle, or lower esophagus, or the gastroesophageal junction.
2. The experimental group received NCT (including perioperative chemotherapy or preoperative chemotherapy).
3. The control group received NCRT.
4. The study reported overall survival (OS) or progression-free survival (PFS) as the primary outcome measure. Secondary outcomes include at least one of the following: pCR (pathological complete remission), R0 resection (no tumor cells within 1 mm of any resection margin), short-term postoperative mortality, treatment-related grade 3–5 adverse events, patterns of recurrence.
5. The study design was a randomized controlled trial (RCT).

Exclusion criteria were applied as follows:

1. Studies were excluded if the full text was unavailable.
2. The outcome of interest was not reported.
3. When multiple articles reported the same clinical trial, only the most comprehensive or up-to-date article was included.

### Assessment of quality

Assessment of quality of included studies adopted the Cochrane Handbook methodology to evaluate potential biases, including randomization, allocation concealment, blinding, incomplete data, and selective reporting. Each assessment criterion was classified as “low risk”, “high risk”, or “unclear risk” based on predefined methodological standards. All evaluation results were systematically recorded and subsequently visualized through graphical representations generated using Review Manager 5.4 software (The Cochrane Collaboration).

### Data extraction

Two investigators independently performed data extraction using a predesigned form. This form included the following elements:

1. Study characteristics: authors, publication year, country, and clinical trial registration number.
2. Participant characteristics: gender, age, tumor stage, stamina, and number of subjects.
3. Treatment details: therapeutic regimen, type of surgery, follow-up duration.
4. Outcome measures: OS and PFS data, expressed as hazard ratio (HR) with corresponding 95% confidence interval (95%CI). Additional secondary outcome endpoints included: pCR, R0 resection, short-term postoperative mortality, treatment-related grade 3–5 adverse events, and recurrence patterns encompassing local recurrence only, distant recurrence only, or both.

### Statistical analysis

Statistical analysis of primary outcomes (OS and PFS) and secondary outcomes was performed using STATA 12.0 software. HRs with 95%CIs were employed to evaluate the efficacy difference between NCT and NCRT in esophageal cancer (including gastroesophageal junction cancer) patients. An HR < 1 indicated superior outcomes in the experimental group (NCT), while an HR > 1 favored the control group (NCRT). Statistical analysis of secondary endpoints used RR (relative risk) and 95% CI to evaluate dichotomous variables. For the favorable outcomes of pCR and R0 resection, an RR < 1 indicated superiority of the control group (NCRT), while an RR > 1 suggested superiority of the experimental group (NCT). For adverse outcomes such as short-term postoperative mortality, treatment-related grade 3–5 adverse events, and recurrence patterns, an RR < 1 indicated superiority of the experimental group (NCT), while an RR > 1 suggested superiority of the control group (NCRT). The chi-square test and I^2^ were used to assess the heterogeneity of the studies, with I^2^ ≥ 50% or p ≤ 0.05 considered significant, and I^2^ < 50% or p > 0.05 considered low. Given the inherent heterogeneity among studies (e.g., variations in therapeutic regimens, surgical procedures, tumor stage, and ethnicities), a random-effects model was applied for pooled analyses to enhance the reliability of the results. Sensitivity analysis using the leave-one-out method was conducted to assess the robustness of results and mitigate the excessive influence of a single outcome measure. Begg’s rank correlation test was used to assess publication bias. Subgroup analyses may be performed based on sample characteristics, including tumor histology and pharmacotherapeutic regimens. All statistical tests were two-sided, and p < 0.05 was considered statistically significant.

It must be emphasized that our study was a meta-analysis of superiority trials, whose core objective was to demonstrate that the experimental group’s therapy was superior to the control group, rather than a non-inferiority trial (which aimed to prove that the experimental group’s efficacy was not inferior to the control group). This study concluded whether the experimental group was superior based on whether the difference was statistically significant. It could not prove that the experimental group was non-inferior to the control group unless a non-inferiority margin had been predefined and demonstrated.

## Results

### Studies selection

**Figure 1:**
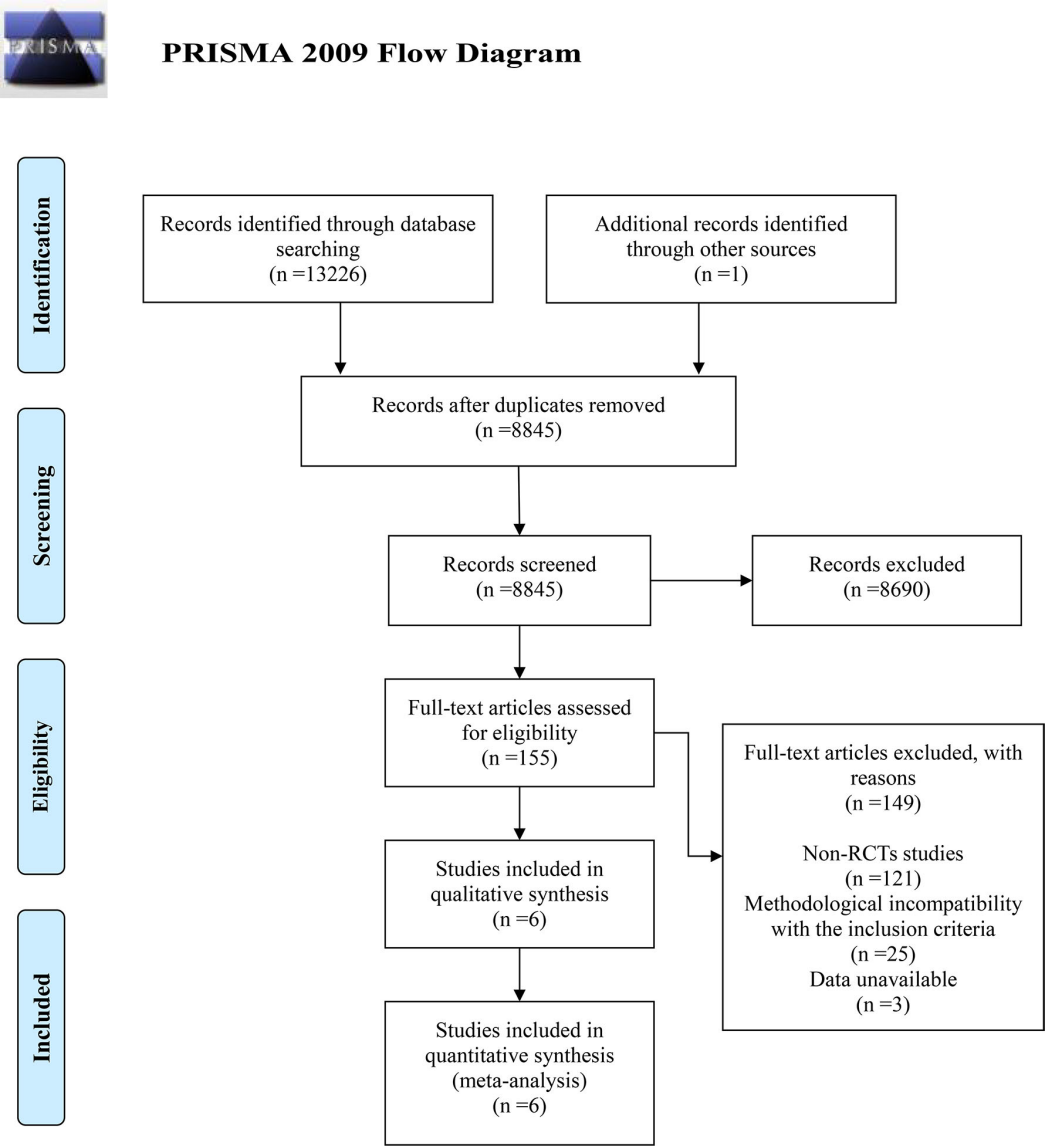
Flow chart representing details of the study selection.

A total of 13,226 results are obtained from direct searches in four electronic databases based on the previously established strategy. Additionally, one article is retrieved from the references, resulting in a total of 13,227 results from the initial search. After removing duplicates, 8,845 results remain. Through reviewing the titles and abstracts, 8,690 articles unrelated to the target topic are excluded. After reading the full texts of the remaining 155 articles, 149 are excluded for the following reasons: 121 are non-RCTs, 25 have methodological issues, and 3 have unstatistical data. Ultimately, 6 articles are included (6, 10, 14–17). The search process is detailed in **Figure 1**.

### Study characteristics

The six included RCTs encompass 1,763 patients from China, Germany, Ireland, Japan, and Sweden, with ages ranging from 18 to 86 years. Publication years span from 2017 to 2025. Among these studies, two focus on ESCC, three on EAC, and one includes both histological types. The predominant chemotherapy regimens consist of cisplatin with fluorouracil (PF regimen). Some studies utilize the fluorouracil, leucovorin, oxaliplatin, and docetaxel (FLOT regimen). Radiotherapy is typically administered at a dose of 41.4 gray (Gy), delivered in 20 to 23 fractions. Common surgical approaches in the study included transesophageal resection, minimally invasive surgery, and gastrectomy. The interval between neoadjuvant therapy and surgery was predominantly 4 to 8 weeks, with an allowable range of 3 to 10 weeks to accommodate logistical factors. The follow-up period ranged from 36 to 60 months. Detailed data are presented in **Table 1** and **Supplementary Table S1**.

**Table 1:** Characteristics of all the studies included in this meta-analysis.

Author	Year	Country	Clinical trial number	Intervene					Control					Types of cancer
				Chemotherapy				No. of patients	Preoperative chemotherapy		Radiotherapy		No. of patients	
				Preoperative		Postoperative								
				Regimen	Course	Regimen	Course		Regimen	Course	Dose (Gy)	Frequency		
Tang, H.	2023	China	NCT03001596	paclitaxel + cisplatin	2	–	–	132	paclitaxel + cisplatin	4	40	20	132	ESCC
Hoeppner, J.	2025	Germany	NCT02509286	FLOT	4	FLOT	4	221	CROSS	5	41.4	23	217	EAC
Reynolds, J. V.	2023	Ireland	NCT01726452	FLOT	4	FLOT	4							
				epirubicin + cisplatin/oxaliplatin + fluorouracil/capecitabine	3	epirubicin + cisplatin/oxaliplatin + fluorouracil/capecitabine	3	184	paclitaxel + carboplatin	5	41.4	23	178	EAC
Kato, K.	2024	Japan	jRCTs031180202	fluorouracil + cisplatin + ciprofloxacin/levofloxacin	2	–	–	199	fluorouracil + cisplatin	2	41.4	23	200	ESCC
Stahl, M.	2017	Germany	NA	folinic acid/5-fluorouracil + cisplatin	2.5	–	–	59	folinic acid/5-fluorouracil + cisplatin + cisplatin + etoposide	3	30	15	60	EAC
von Döbeln, G. A.	2019	Sweden	NCT01362127	cisplatin/oxaliplatin/carboplatin + 5-fluorouracil	3	–	–	91	cisplatin/oxaliplatin/carboplatin + 5-fluorouracil	3	40	20	90	EAC, ESCC

No, number; Gy, Gray; FLOT, fluorouracil, leucovorin, oxaliplatin, docetaxel; CROSS, carboplatin, paclitaxel, radiotherapy; ESCC, esophageal squamous cell carcinoma; EAC, esophageal adenocarcinoma.

### Quality assessment

The Cochrane Handbook is used to assess the quality of each study. Using three indicators—low risk, high risk, and unclear risk—the quality of studies is evaluated across domains, including randomization, allocation concealment, blinding, incomplete data, and selective reporting. Most studies fail to report whether allocation concealment was implemented or whether outcome assessors were blinded, resulting in uncertain risk. Most studies do not blind participants or primary investigators, resulting in a high risk of performance bias. While most studies demonstrate low risk across most domains, Stahl,M.’s study, which was terminated early, exhibited high risk for follow-up bias and reporting bias. Details are shown in **Supplementary Figure S1** and **S2**.

### Analysis of main results

**Figure 2:**
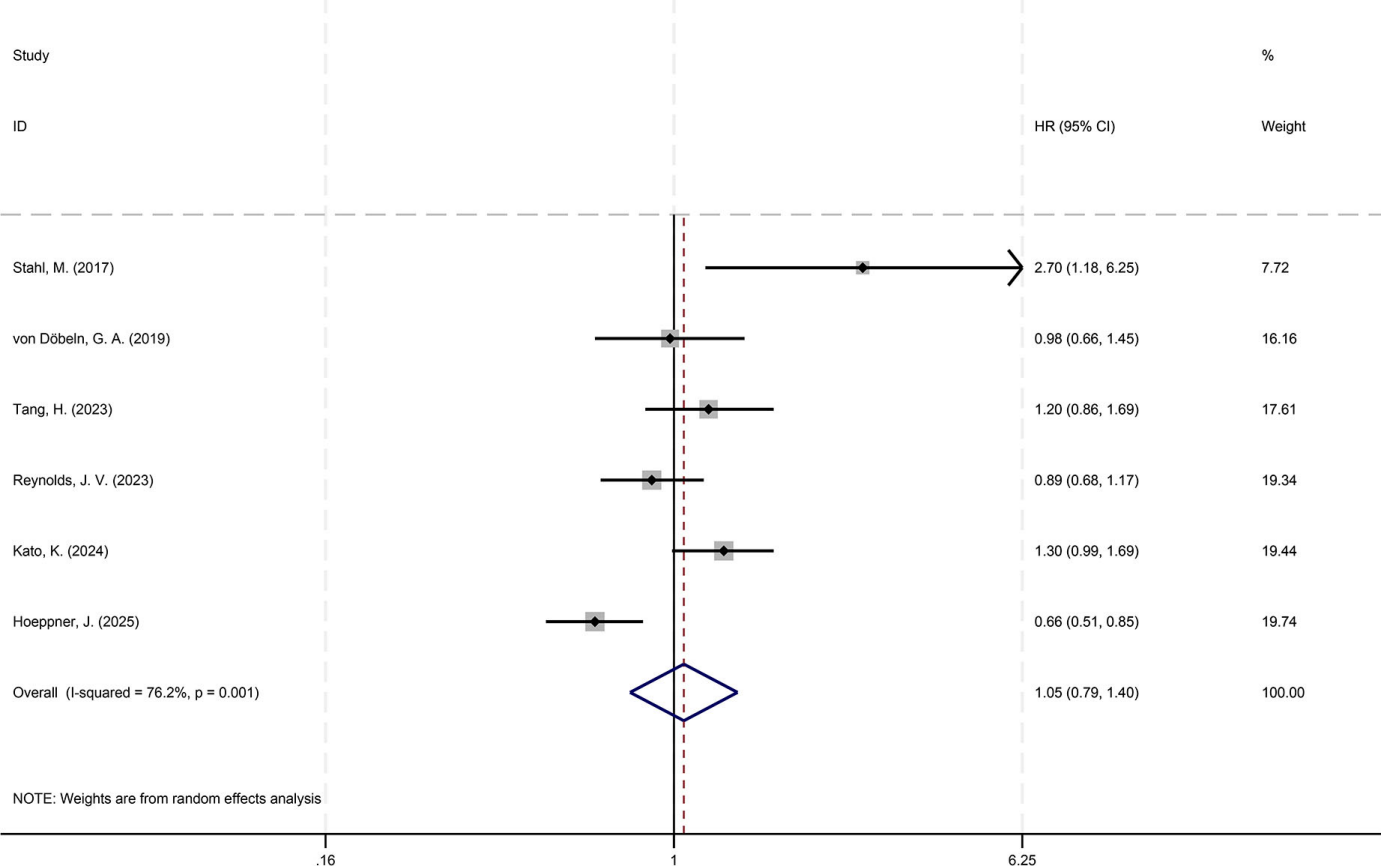
Forest plot showing the effect of NCT and NCRT on PFS (p=0.718).

**Figure 3:**
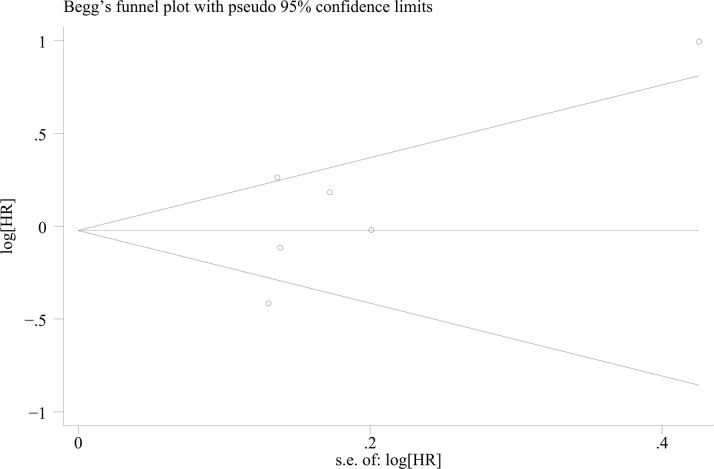
Begg’s funnel plot showing publication bias of PFS (p=0.260).

**Figure 4:**
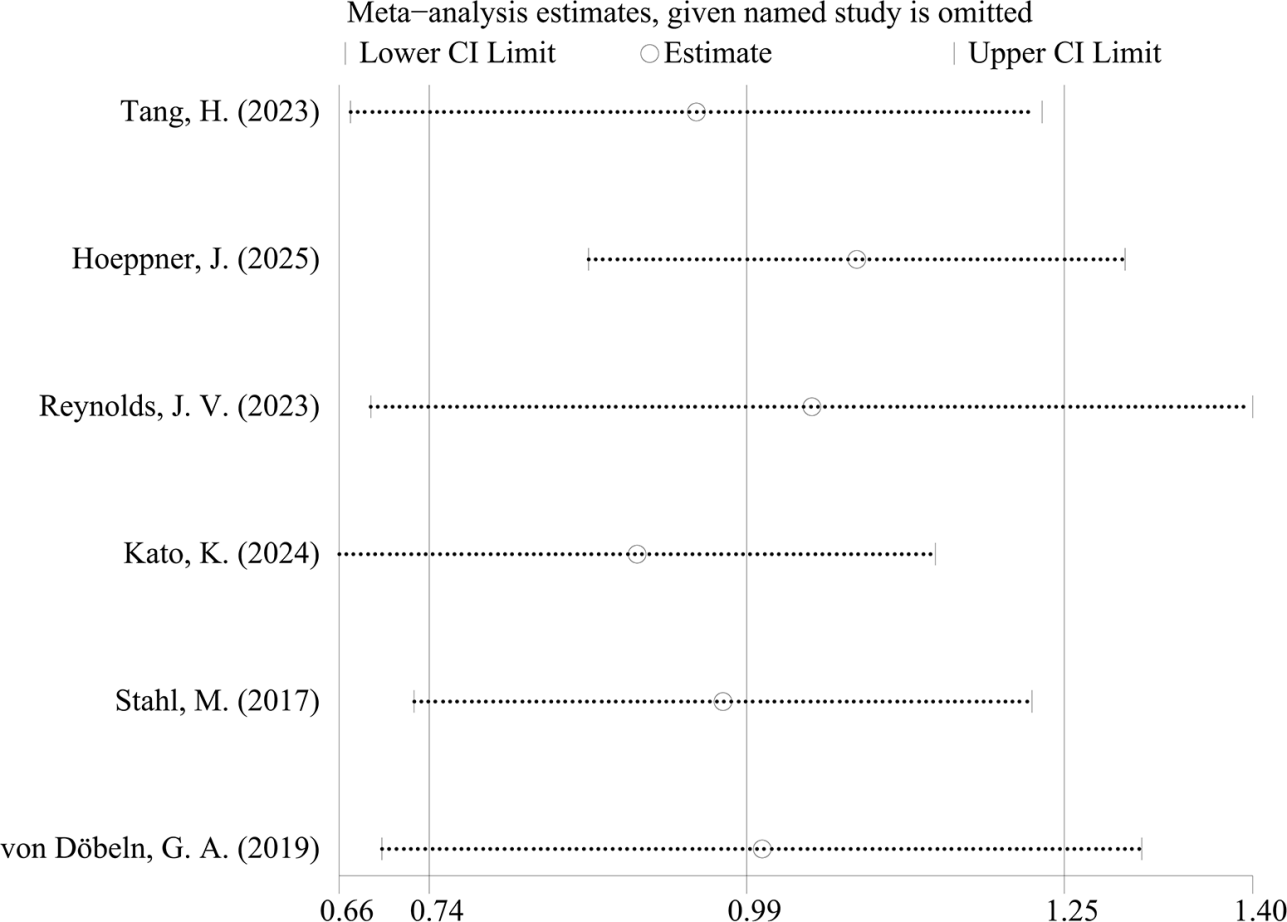
Plot of sensitivity analysis of PFS.

Analysis of HRs and their 95%CIs for PFS and OS across the six studies yields the following results. For the pooled analysis of PFS, the results under the random-effects model indicate no statistically significant difference between the experimental group and the control group (HR = 1.05, 95%CI: 0.79-1.40; p=0.718, I²=76.38%, τ²=0.0892). However, substantial heterogeneity is observed (I²=76.2%) (**Figure 2**). Publication bias assessment for PFS shows no significant bias (p=0.260) (**Figure 3**). Sensitivity analysis with sequential exclusion of each study shows stability of the results (**Figure 4**).

**Figure 5:**
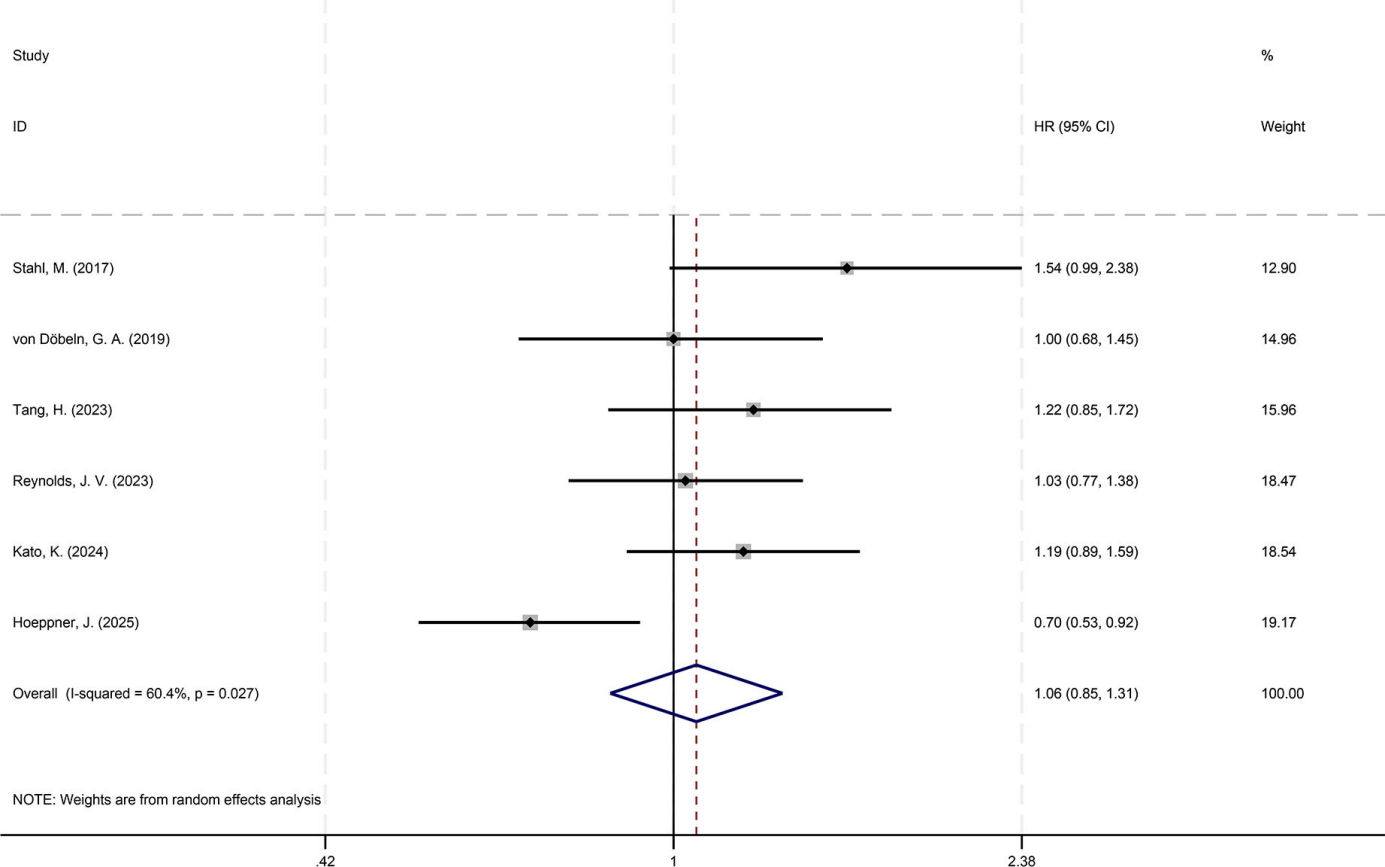
Forest plot showing the effect of NCT and NCRT on OS (p=0.603).

**Figure 6:**
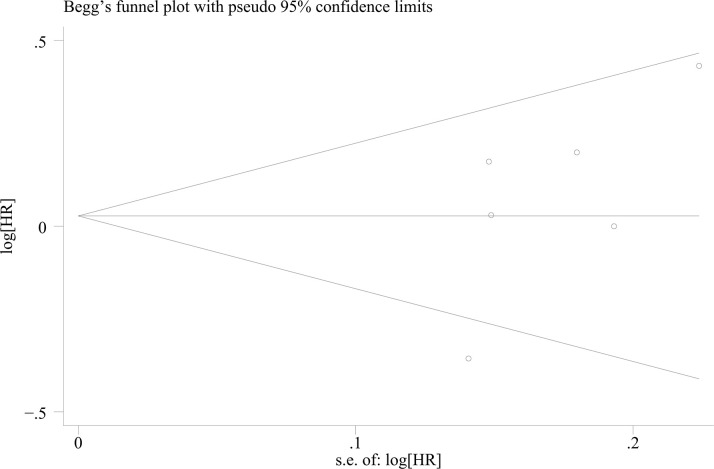
Begg’s funnel plot showing publication bias of OS (p=0.452).

**Figure 7:**
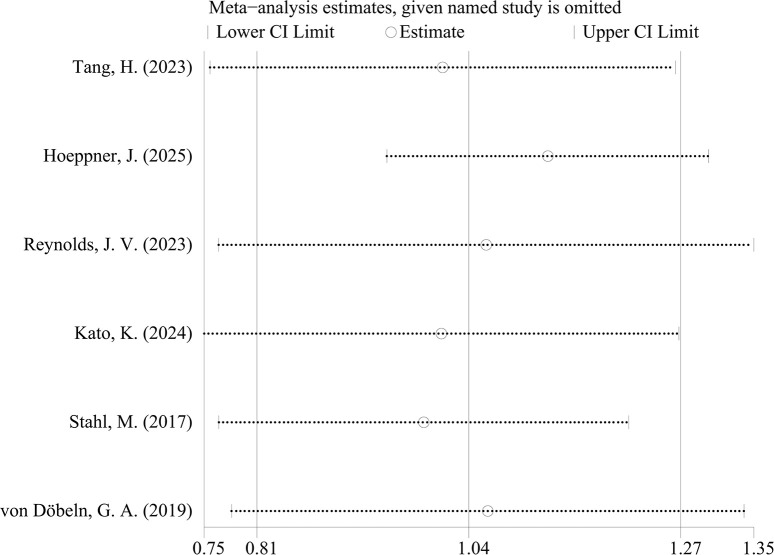
Plot of sensitivity analysis of OS.

For the pooled analysis of OS, the results under the random-effects model similarly indicate no statistically significant difference between the experimental group and the control group (HR = 1.06, 95%CI: 0.85-1.31; p=0.603, I²=59.53%, τ²=0.0411). Considerable heterogeneity is present (I²=60.4%) (**Figure 5**). Publication bias assessment for OS reveals no significant bias (p=0.452) (**Figure 6**). Sensitivity analysis, performed by excluding each study one at a time, confirms the stability of the results (**Figure 7**).

The pooled analysis of secondary outcomes under the random-effects model reveal no significant differences between the control and experimental groups in the following aspects: pCR (RR = 0.35, 95% CI: 0.11-1.04; p=0.06), short-term postoperative mortality (RR = 0.55, 95% CI: 0.27-1.11; p=0.10), grade 3–5 adverse events (RR = 1.02, 95% CI: 0.75-1.39; p=0.89), distant recurrence only (RR = 0.82, 95% CI: 0.65-1.04; p=0.10), both types of recurrence (RR = 1.01, 95% CI: 0.70-1.47; p=0.94). However, the control group (NCRT) demonstrates superiority over the experimental group (NCT) in terms of R0 resection (RR = 0.93, 95% CI: 0.88-0.99; p=0.02) and also shows superiority in terms of local recurrence only (RR = 1.38, 95% CI: 1.07-1.78; p=0.01). Details are shown in **Table 2**.

**Table 2:** Short-term outcome analysis.

Subgroup	No. of studies	RR	95%CI	P	Heterogeneity	
					I^2^	P
pCR	5	0.35	(0.11-1.04)	0.06	86%	<0.001
R0 resection	6	0.93	(0.88-0.99)	0.02	73%	0.002
Short-term postoperative mortality	4	0.55	(0.27-1.11)	0.10	0%	0.44
Treatment-related grade 3-5 adverse events	3	1.02	(0.75-1.39)	0.89	67%	0.05
Recurrence pattern						
Local recurrence only	6	1.38	(1.07-1.78)	0.01	0%	0.79
Distant metastasis only	5	0.82	(0.65-1.04)	0.10	32%	0.21
Both	5	1.01	(0.70-1.47)	0.94	40%	0.15

pCR, pathological complete remission; R0, no tumour cells within 1 mm of any resection margin; RR, relative risk; CI, confidence interval.

## Discussion

This meta-analysis incorporates six RCTs comparing the efficacy of NCT versus NCRT during the perioperative period in patients with locally resectable esophageal cancer (including gastroesophageal junction cancer). By assessing OS and PFS across both groups, the pooled estimates do not demonstrate a significant survival benefit for NCRT over NCT. Similarly, pooled estimates for secondary outcomes show no significant differences between NCT and NCRT in pCR, short-term postoperative mortality, treatment-related grade 3–5 adverse events, distant recurrence alone, or both types of recurrence. However, patients receiving NCRT demonstrated superior rates of R0 resection and lower local recurrence rates compared to the NCT group, underscoring that NCRT retains a significant advantage in local tumor control.

Esophageal cancer is a highly malignant, aggressive disease with poor prognosis, presenting significant treatment challenges. For locally resectable esophageal cancer, treatment strategies have evolved from initial approaches of radiotherapy alone or surgery alone to multimodal therapies combining surgery with chemotherapy or radiotherapy to enhance efficacy. Subsequently, based on Van Hagen’s research, NCRT significantly improved OS compared to surgery alone (HR = 0.67, 95%CI: 0.50-0.88) (5). Studies by Zhou et al. demonstrated superior OS with NCRT compared to NCT, as evidenced by both meta-analysis (HR = 0.82, 95%CI: 0.72-0.93) and cohort study (HR = 0.60, 95%CI: 0.40-0.91) (11). Furthermore, guidelines published in 2022 recommend NCRT followed by surgery as the standard treatment regimen for locally resectable esophageal cancer (7).

However, an increasing number of studies indicate that for patients with locally resectable esophageal cancer, there is no significant difference in survival outcomes between NCRT and NCT. For example, a European multicenter study demonstrated no difference in OS between patients receiving NCRT and NCT (HR = 1.10, 95%CI: 0.98-1.25) (18). Faron’s network meta-analysis similarly showed no significant difference in OS between the two regimens (HR = 0.90, 95%CI: 0.74-1.09) (12). Among the six RCTs included in our meta-analysis, the earliest study(Stahl et al.) supported NCRT (15), while the most recent study (Hoeppner et al.) favored NCT (14). The remaining four studies observed no significant survival differences (6, 10, 16, 17). The mechanisms by which NCRT is not superior to NCT may be as follows.

First, the evolution of chemotherapy regimens has significantly improved treatment outcomes for locally resectable esophageal cancer. In earlier studies targeting locally resectable esophageal cancer, chemotherapy regimen consisted of two cycles of cisplatin combined with bleomycin (19). As a glycopeptide antibiotic with potent chemotherapeutic activity, bleomycin is no longer used in modern treatment regimens for esophageal cancer due to its potential side effects, including pulmonary fibrosis and neurological deficits (20). Subsequently, the PF regimen became the standard chemotherapy for esophageal cancer (21). However, subsequent studies demonstrated that the triple-drug combination therapy containing cisplatin, fluorouracil, and docetaxel (TPF regimen) significantly improved OS and PFS compared to the dual-drug PF regimen (22, 23), and Kato’s RCT (6) also confirmed this conclusion. Subsequent studies demonstrated that the FLOT regimen offers superior OS compared to TPF (HR = 0.77, 95%CI: 0.63-0.94) (24). Consequently, current guidelines recommend FLOT as the standard perioperative treatment for patients with locally resectable adenocarcinoma (25). Stahl’s study exclusively enrolled patients with gastroesophageal junction adenocarcinoma who received preoperative chemotherapy with fluorouracil or leucovorin plus cisplatin (rather than the currently preferred FLOT regimen) (15). This may stem from the limited efficacy of early chemotherapy regimens, which left room for survival benefits from radiotherapy, thereby explaining the superiority of chemoradiotherapy over chemotherapy alone at that time.

Second, with continuous advancements and maturation in surgical techniques, the rate of radical resection for locally resectable esophageal cancer has gradually increased, while the benefits of radiotherapy have become relatively limited. Early traditional open thoracic and abdominal surgeries yielded unsatisfactory outcomes, characterized by low cure rates, numerous surgical complications, and perioperative mortality rates as high as 10%-30% (26). At that time, radiotherapy filled the therapeutic gap left by surgery. With advances in medical technology, the European Society for Medical Oncology guidelines now recommend thoracic radical resection combined with en bloc bilateral lymph node dissection as the preferred surgical approach for esophageal cancer patients (7). For distal tumors, reconstruction is achieved via a right thoracic and abdominal approach, performing an upper mediastinal esophagogastric anastomosis (Ivor Lewis procedure) through a gastric tube, and for tumors in the middle and upper esophagus, a combined laparoscopic, right thoracic, and neck approach is employed, with reconstruction similar to that for cervical esophageal cancer (McKeown procedure) (7). At high-volume centers with modern surgical capabilities, the R0 resection rate for radical resection of thoracic esophageal cancer can reach approximately 94% (14). Furthermore, many studies demonstrate that minimally invasive esophagectomy (MIE) not only achieves a high R0 resection rate of approximately 97% (16), but also offers patient benefits such as reduced trauma and faster recovery compared to open thoracic esophagectomy (27, 28). Additional research confirms superior efficacy of MIE, with a 18% reduction in 5-year all-cause mortality compared to open surgery (29). These techniques constitute the mainstream surgical approaches in the studies included in this meta-analysis. In contrast, the study supporting NCRT exhibit lower surgical quality standards: the R0 resection rate in Stahl’s study was only about 70%, lower than the resection rates in all other included studies in our meta-analysis (approximately 79%-97%). Furthermore, the interval between neoadjuvant therapy and surgery in this study (only 3–4 weeks) was shorter than in other studies (4-8 weeks). Previous studies indicates that extending the interval between neoadjuvant therapy and surgery in responsive patients improves OS (30). Conversely, an excessively short interval may fail to achieve optimal pathological response, thereby compromising surgical success rates and long-term prognosis. Additionally, an appropriate interval allows patients sufficient recovery time to undergo surgery in a better physiological condition. Subsequent clinical trials employed more refined surgical techniques and strictly adhered to optimal surgical timing, yielding superior treatment outcomes. This may have diminished the radiotherapy gain effect, representing another potential reason why NCRT no longer demonstrates a clear advantage over NCT.

Third, chemotherapy and radiotherapy each possess distinct therapeutic advantages, with OS and PFS influenced by both systemic and local control. NCRT typically improves local control and pCR rates at the cost of increased acute toxicity; NCT, however, is more conducive to systemic control and exhibits different toxicity profiles. The lack of significant differences in OS and PFS across patient populations may reflect a balancing effect between the respective strengths and limitations of these approaches. Esophageal cancer is a highly invasive malignancy. Beyond direct local invasion around the primary tumor, cancer cells readily metastasize distally (31), with distant metastasis being the primary cause of postoperative mortality in esophageal cancer patients (10, 32). Chemotherapy, as a systemic agent, can eliminate distant metastatic lesions. In contrast, radiotherapy can only eliminate local lesions, thereby reducing the local recurrence rate (which is not the primary cause of death), but failing to decrease distant metastasis or eliminate metastatic tumor cells. Therefore, NCRT does not offer a significant difference over NCT in terms of survival benefits. Nevertheless, we must acknowledge the superiority of radiotherapy in achieving local tumor control. Our study’s pooled estimates indicate that NCRT outperforms NCT in achieving R0 resection and reducing local recurrence, suggesting that NCRT may be the preferred option in cases with high local tumor risk.

Fourth, the chemotherapy dose in NCRT is typically lower than that in NCT, thereby limiting its scope and potentially affecting its efficacy. With advances in chemotherapy regimens, the advantages of NCRT over NCT has gradually diminished.

It is noteworthy that a 2025 RCT by Hoeppner et al. was the first to report superior OS and PFS with perioperative chemotherapy versus NCRT, indicating greater efficacy with fewer side effects (14). First, as this remains the only published RCT evaluating perioperative FLOT chemotherapy alone, it suggests the FLOT regimen itself possesses sufficient efficacy. By the way, Reynolds’s study also included the FLOT regimen, but adherence is low (13%) (10). Second, the addition of radiotherapy in neoadjuvant therapy may reduce treatment compliance and weaken overall efficacy due to associated side effects such as organ dysfunction and impaired tissue regeneration (33). Finally, it should be noted that Hoeppner’s study exclusively enrolled patients with EAC. Whether similar conclusions apply to patients with ESCC requires validation in future RCTs.

The strength of this report lies in being the first meta-analysis to include only RCTs and to conclude that NCT and NCRT are equivalent in the perioperative treatment of locally resectable esophageal cancer. The included studies are of high quality and provide high-quality evidence. Sensitivity analysis using the stepwise method demonstrated stable results, indicating that the high/uncertain risk present in the included articles does not affect the reliability of our pooled OS/PFS estimates. When the treatment effect is the same, if NCT is used instead of NCRT, it will not only reduce the burden of patients but also save medical resources.

However, this paper also has some shortcomings:

1. Due to the limited number of included studies (only 6 RCTs), the test’s efficacy is constrained. Although publication bias was assessed, the risk of publication bias cannot be entirely ruled out. Furthermore, it is insufficient to detect small differences in benefits for OS and PFS.
2. Due to insufficient extractable data, trial-level pooled data rather than individual patient data were used, precluding more refined subgroup analyses. This limitation restricted our ability to assess the impact of tumor histology, patient ethnicity, and specific chemoradiotherapy regimens on the efficacy of the two neoadjuvant treatments.
3. The included studies exhibited significant heterogeneity in clinical and methodological aspects, which may have impacted the reliability of the results. Key factors included: different histological types of esophageal cancer, varying geographic regions, distinct healthcare systems, differing chemotherapy regimens, variations in radiotherapy doses, techniques, and surgical standards, as well as perioperative care. These factors may influence the relative benefits of NCRT versus NCT. For instance, squamous cell carcinoma may derive greater benefit from radiotherapy than adenocarcinoma, and modern FLOT regimens show significant efficacy differences compared to older NCT protocols. Stahl’s study exhibited relatively high heterogeneity, enrolled a smaller patient cohort, and employed the lowest radiotherapy dose (30 Gy) and fractionation (15 fractions) (15). This may have reduced radiotherapy-related side effects, leading to superior outcomes in the NCRT group compared to NCT. Furthermore, Stahl’s study featured the shortest interval between neoadjuvant therapy and surgery (3–6 weeks), potentially introducing a bias toward improved surgical outcomes in the overall patient population (15). This bias may have amplified the apparent synergistic advantage of NCRT, leading to results that disproportionately favor NCRT superiority.
4. As a meta-analysis of efficacy trials, our pooled effect size did not demonstrate statistical differences. This only proves the absence of superiority, not equivalence or non-inferiority.

Overall, we found that NCRT did not demonstrate a clear advantage over NCT in terms of OS and PFS, while showing superiority in achieving R0 resection and reducing local recurrence. This suggests that NCRT should not be entirely abandoned but rather emphasizes the need to support individualized decision-making. In certain scenarios, such as for patients with locally high-risk ESCC, local tumor control is more critical for survival and prognosis, potentially warranting continued prioritization of NCRT. In other cases, chemotherapy alone may be more appropriate—for instance, the FLOT regimen used in Western settings for NCT in patients with EAC.

## Conclusion

This study indicates that in the perioperative management of locally resectable esophageal cancer (including gastroesophageal junction cancer), our data have not yet demonstrated a clear survival advantage for NCRT over NCT (lack of statistical significance does not imply equivalence), particularly in the context of mixed histological types and treatment regimens.

## Supplementary material

Supplementary Figure 1 Risk of bias assessment in included studies.

Supplementary Figure 2 Summary of risk of bias across studies.

Supplementary Table 1 Characteristics of the studies included in this meta-analysis.
