# Supplementary figures and images for: A meta-analysis of neoadjuvant chemotherapy versus neoadjuvant chemoradiotherapy for locally resectable esophageal cancer based on RCTs
Source: Front Oncol. 2026 Feb 12;16:1728150. doi: 10.3389/fonc.2026.1728150 (PMC12935649; doi:10.3389/fonc.2026.1728150)

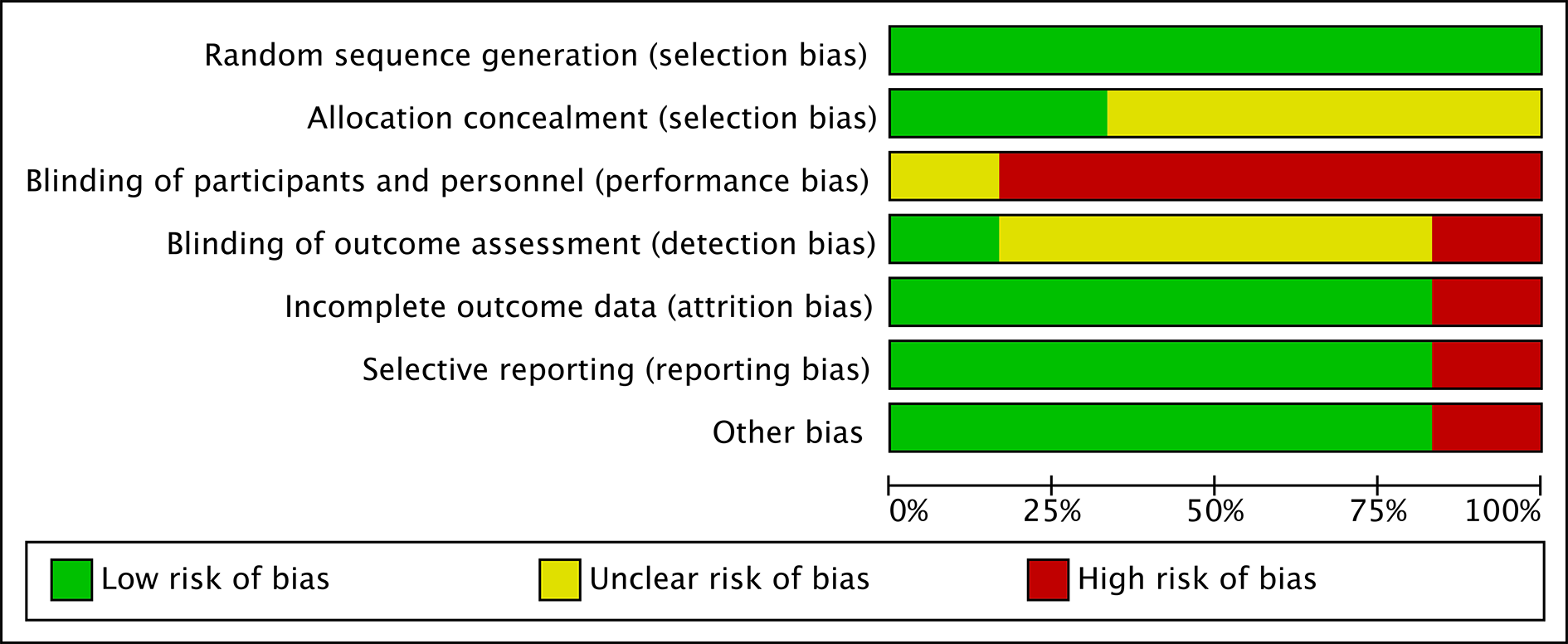

Supplement: Supplementary Figure 1 — Risk of bias assessment in included studies. [file Image1.tif]

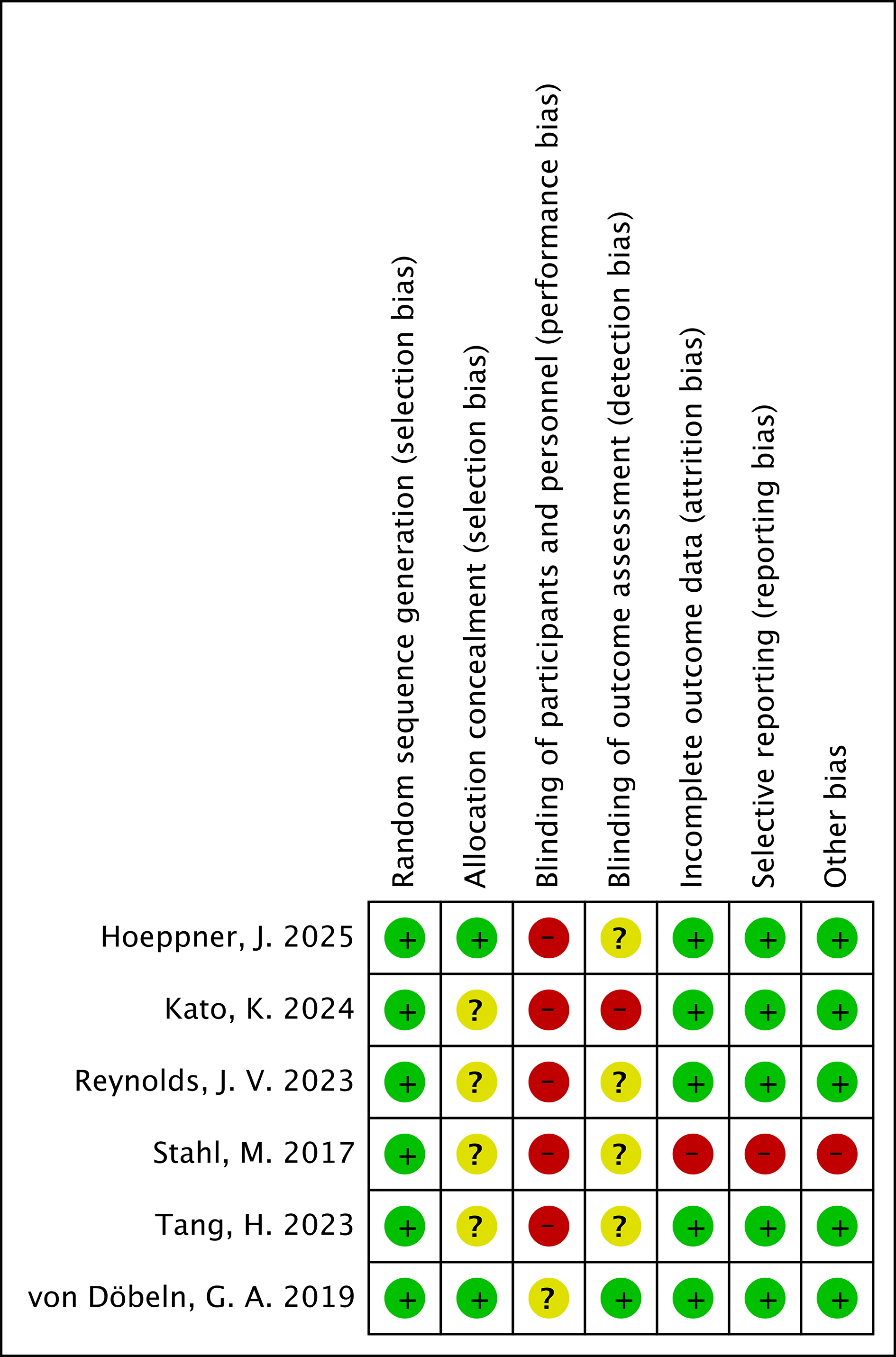

Supplement: Supplementary Figure 2 — Summary of risk of bias across studies. [file Image2.tif]
