# Supplementary material for: A meta-analysis of neoadjuvant chemotherapy versus neoadjuvant chemoradiotherapy for locally resectable esophageal cancer based on RCTs
Source: Front Oncol. 2026 Feb 12;16:1728150. doi: 10.3389/fonc.2026.1728150 (PMC12935649; doi:10.3389/fonc.2026.1728150)
Supplement: Supplementary Table 1 — Characteristics of the studies included in this meta-analysis. [file Table1.docx]

**Supplementary Table 1. Characteristics of all the studies included in the meta-analysis.**

| Author | Year | Recruitment year | Follow-up time | Age (year) | Tumor location | Type of surgery | Interval between neoadjuvant therapy and surgery |
| --- | --- | --- | --- | --- | --- | --- | --- |
| Tang, H. | 2023 | 2017-2019 | 3 year | 18-75 | upper thoracic,  middle thoracic,  lower thoracic | minimally invasive esophagectomy | 4-8 weeks |
| Hoeppner, J. | 2025 | 2016-2020 | 55 month | 30-86 | oesophagus,  oesophagogastric junction | transthoracic esophagectomy, extended gastrectomy,  esophagogastrectomy | 4-8 weeks |
| Reynolds, J. V. | 2023 | 2013-2020 | 38.8 month | 63.8 (median) | esophagogastric junction | en-bloc two-stage transthoracic resection, minimally invasive en-bloc, en-bloc three-stage transthoracic resection, extended total gastrectomy and mediastinal anastomosis, extended total gastrectomy and thoracic anastomosis, trans-hiatal oesophagectomy | 3-10 weeks |
| Kato, K. | 2024 | 2012-2018 | 50.7 month | 30-75 | upper thoracic,  middle thoracic,  lower thoracic | total or subtotal thoracic oesophagectomy and regional lymphadenectomy with right thoracotomy | <8 weeks |
| Stahl, M. | 2017 | 2000-2005 | 5 year | ≤70 | esophagogastric junction | transthoracic esophagectomy, transhiatal esophagectomy,  gastrectomy | 3-6 weeks |
| von Döbeln, G. A. | 2019 | 2006-2013 | 5 year | 37-75 | esophagus,  gastroesophageal junction | Ivor–Lewis resection, three-stage esophagectomy,  transhiatal esophagectomy, total gastrectomy | 4-6 weeks |
